# Ketal-Modified Cellulose as a Biodegradable Bioplastic

**DOI:** 10.1021/acsomega.5c07631

**Published:** 2025-11-14

**Authors:** Kyle E. Broaders, Elizabeth Kuehne, Abigail C. Bowden, Maxine R. Fraser

**Affiliations:** Department of Chemistry, 7397 Mount Holyoke College, South Hadley, Massachusetts 01075, United States

## Abstract

Polymeric plastic materials pervade every part of modern society. The vast majority of these plastics are petroleum-based; their production generates over 2 gigatons of CO_2_ equivalent annually, and their waste biodegrades extraordinarily slowly. Thus, there is an urgent need to transition away from nonrenewable nonbiodegradable plastics. Biologically derived polymers hold potential to produce plastics with drastically reduced environmental impact, during both their production and waste remediation. Cellulose esters and ethers are among the oldest successful renewable plastics, but they can suffer limitations related to their processability and degradability. We report on the homogeneous phase chemical modification of cellulose to form methoxy isopropylidine acetal-modified cellulose, MiP-Cel. Chemical and materials characterization reveals a high degree of substitution and excellent solution processability. MiP-Cel forms transparent, smooth, freestanding films, which are measured for optical clarity and hydrophobicity. Finally, the pH-dependent degradation of MiP-Cel-derived materials is assessed.

## Graphical abstract

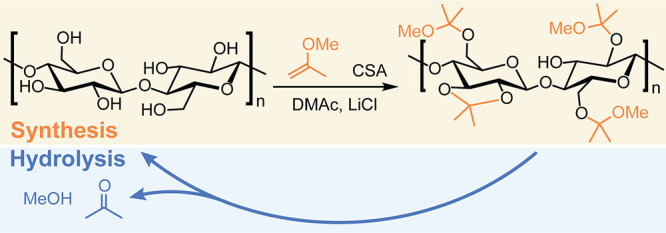

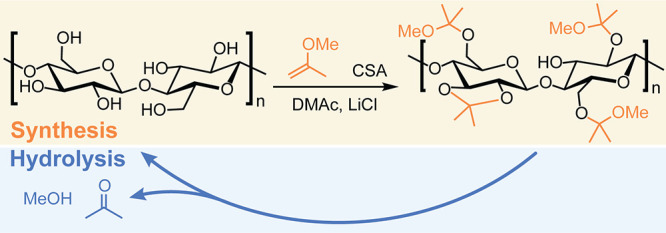

## Introduction

The production of polymeric plastics has grown to over 460 million tons every year, with an average yearly growth of over 8%.^1^ The vast majority of these plastics are made of just a small number of petroleum-derived polymers, with just poly­(ethylene) and poly­(propylene) making up over 57% of global plastics production.^2^ Producing these polymers generates atmospheric emissions that are the equivalent of over 2.25 gigatons of carbon dioxide annually.^3^ Beyond the cost in producing these polymers, the eventual fate of plastic waste has been the subject of increasing scrutiny as awareness grows of both concentrated large plastic pollution exemplified by the Great Pacific Garbage Patch and in pervasive but diffuse pollution in the form of microplastics.^4,5^ Given these factors, there is an urgent need to transition away from nonrenewable nonbiodegradable plastics. Biologically derived polymers hold potential to produce plastics with drastically reduced environmental impact, during both their production and waste remediation.^6^

The most commonly used bioderived biodegradable plastic materials are made from poly­(lactic acid), PLA.^7^ This thermoplastic material has properties similar to those of petroleum-based polymers like poly­(styrene) and poly­(ethylene terephthalate), but its feedstock, lactide, can be prepared from the fermentation of carbohydrates from crop sources such as corn and sugar cane. Life-cycle analysis (LCA) studies are widely varied on the relative environmental benefits of using PLA in place of petroleum-derived materials, largely due to emissions related to large-scale fermentation.^8,9^ Polyhydroxyalkanoates are another class of bioplastics that are rising in prominence. They are directly produced in microorganisms and have a range of material properties that can be manipulated through metabolic engineering.^10^ Although improvements have been made in recent years, production costs limit the growth in usage of these polymers.^11,12^

Cellulose-based materials hold the potential to expand the scope of viable biodegradable materials. Cellulose is well appreciated to be the world’s most abundant polymer, being produced as an important structural molecule in plants, algae, tunicates, and some species of bacteria.^13–15^ It is additionally naturally degradable via cellulase activity held by a variety of microbes and fungi.^16,17^ Cellulose is a linear β-(1→4) linked polymer of d-glucose. It is able to make strong hydrogen bonds, leading to the formation of linear chains that bundle together into microfibrils made of 20–200 individual polymer chains. In native cellulose, these crystalline regions are generally interspersed with amorphous regions to form cellulose fibers. Microcrystalline cellulose is often used in the preparation of cellulosic biomaterials; it is formed through partial acidic hydrolysis of cellulose, removing the amorphous regions and reducing the degree of polymerization (DP) to approximately 100.^18^

Cellulose has a long history as a feedstock for biomaterials. Cellophane, Celluloid, and Acetate, made from regenerated cellulose, nitrocellulose, and cellulose acetate, respectively, were some of the earliest widespread plastics. Carboxymethylcellulose is still widely used across a wide range of industries,^19^ as are cellulose ethers like methylcellulose and hydroxypropylmethyl cellulose.^20^ While less explored than esters and ethers, acetals may hold promise as new functional modifications to cellulose. Due to their inherently divalent structure, each acetal can substitute one or two hydroxyls with variably hydrophobic groups. Acetals are chemically inert to nearly all conditions except mildly acidic aqueous environments, providing a potential avenue for triggered biodegradation or the development of smart materials.^21–23^ This strategy has been applied extensively through ketal-type acetals in dextran,^24^ as well as cyclodextrin^25^ and maltodextrin.^26^ A significant feature in all of these applications is the ability to tune processability and degradation rate by modulating the amounts of linear and cyclic acetals.^27^ Cyclic acetals are formed from the reaction of a linear acetal with a neighboring hydroxyl group under kinetic conditions.^28^ Materials with a higher degree of substitution (DS, modifications per anhydroglucose unit, AGU) feature slower degradation under mildly acidic aqueous hydrolysis.

Although few in number, there are reports of acetal-modified cellulose-based materials. Early reports featured modification with acetals formed by acid-catalyzed reaction with aldehydes in DMAc/LiCl, which led to water-soluble polymers with DS values generally less than 1.0.^29^ More recently, Wurm and co-workers reported the use of aliphatic aldehydes to prepare hydrophobic cellulose-acetal derivatives under thermodynamic conditions. These featured DS greater than 2.0 and could be used to form biodegradable materials. Degradation at reduced pH led to the release of cellulose along with the original aldehyde.^30^

Motivated by the growing need for new biomaterial-based plastics and inspired by past work, we sought to generate a new cellulose-based material based on ketal-type acetals. We report here the synthesis and characterization of MiP-Cel, a hydrophobic cellulose derivative featuring methoxyisopropyl and isopropylidene ketals. The material is highly substituted and freely soluble in organic solvents but can be reverted into cellulose and benign acetone and methanol upon acidic hydrolysis. The material features excellent solution processability and can be used to fashion highly transparent free-standing films. We anticipate that this material may form the basis of an array of acid-responsive biodegradable materials.

## Materials and Methods

### Materials

Chemicals were purchased from Oakwood Chemical, Sigma-Aldrich, or Acros Organics and used without further purification. Compost was produced in an Earth Machine backyard composter and collected from the lower harvest door. Nuclear magnetic resonance (NMR) experiments were performed on a Bruker Avance 400 MHz spectrometer. Infrared Spectroscopy was performed on a Bruker Alpha FTIR-ATR spectrometer. X-ray diffraction was carried out using a Rigaku MiniFlex 600. Unless otherwise stated, all reactions were carried out under an inert atmosphere of argon.

### Dissolution of Cellulose

Microcrystalline cellulose (MCC) was completely dissolved using a modified version of DuPont’s protocol.^31^ Briefly, MCC (1.00 g) was stirred in H_2_O (15 mL) for 1 h. Water was removed by filtration, and the MCC was resuspended in MeOH (15 mL) for 45 min. Filtration and resuspension in MeOH were then repeated a second time. MeOH was exchanged with DMAc in a similar process: two sequential filtrations and resuspensions in DMAc (15 mL) for 30 min each. This activated MCC was added to a solution of dry LiCl (2.5 g) in anhydrous DMAc (30 mL) at 40 °C and stirred until a clear solution was achieved.

### Synthesis of MiP-Cel

To the solution of cellulose (1.00 g, 6.16 mmol AGU) in LiCl/DMAc (8.2%, 30 mL) prepared above was added 2-methoxypropene (3.55 mL, 37.0 mmol) followed by camphorsulfonic acid (CSA, 0.062 mmol, 14 mg). After 1 h, the reaction was quenched by the addition of 2 drops of triethylamine. MiP-Cel was isolated by dropwise precipitation into aqueous NaHCO_3_ (5%, 150 mL) and filtration through a 0.45 μm nylon filter. Crude MiP-Cel was twice purified by precipitation: the crude material was dissolved in warm THF (50 mL), filtered through Celite, and precipitated into hexanes (150 mL). Isolation by filtration and drying in vacuo yielded pure MiP-Cel as a white powder (0.81 g, 42.5% yield). Calculation details related to the degree of modification can be found in the Supporting Information.

### Analysis of MiP-Cel Substitution by Degradation

MiP-Cel (16.47 mg) was added to a clean NMR tube and suspended in D_2_O (0.75 mL, acidified to pD 0 using DCl). The suspension was allowed to react at rt until it became uniformly translucent with no visible solids (∼2 min), and the resulting products were analyzed via ^1^H NMR to determine the composition of the polymer. Repeat acquisition of the spectrum was carried out after 1 h to confirm complete hydrolysis. Relative concentrations of acetone and methanol were measured using integrations normalized to an internal standard, 4,4-dimethyl-4-silapentane-1-sulfonic acid (DSS). Calculation details can be found in the Supporting Information.

### Quantitative ^13^C NMR

To a solution of MiP-Cel (20 mg) dissolved in DMSO-d6 (0.75 mL) was added Cr­(acac)_3_ (3 mg, 9 μmol) as a T1 relaxing agent.^32^ The Bruker zgpg pulse program featuring a 90° pulse and broadband ^1^H decoupling was used with a 3.0 s relaxation delay.

### Film Preparation

A solution of MiP-Cel (75 mg) in THF (1.0 mL) was added via Pasteur pipet to a clean glass slide and spread by using an adjustable film applicator (LianDu-US). Films were allowed to dry in the air for at least 15 min. After drying, the films could be lifted by using a single-edged razor blade and forceps. Film thickness was assessed by using a commercial coating thickness gauge (VVV-Group), and tensile properties were assessed by using a Universal Testing Machine (Instron).

### SEM Imaging

Ac-Cell fiber and films were placed under liquid nitrogen until fully frozen, then picked up with tweezers and broken to create sharp edges. Samples were affixed to carbon tape, sputter-coated with 4 nm Au, and imaged at 1–2 kV. Analysis was carried out using FIJI.^33^

### Bulk Film Degradation

Films were prepared by dropcasting MiP-Cel solution in THF (∼0.1 mL, 90 mg/mL) onto preweighed glass coverslips and dried in a vacuum oven at 50 °C. After recording initial masses, the supported films were placed in 6-well plates and submerged in neutral buffer (0.1 M H_2_PO_4_, pH 7.4, 22 °C), acidic buffer (0.1 M acetate, pH 5.0, 22 °C), or home compost (35 °C). At each time point, films were gently rinsed with ddH_2_O, dried in a vacuum oven at 50 °C for 1 h, and then weighed. Films were then submerged in fresh buffer or returned to the compost.

## Results and Discussion

In analogy to the preparation of Ac-Dex,^27^ Ace-Dex,^34^ and SpAc-Dex,^35^ acetal-modified cellulose was prepared by camphorsulfonic acid-catalyzed addition of an enol ether (Scheme 1). To facilitate this solution-phase reaction, microcrystalline cellulose (MCC) was first activated with water and then solvent exchanged and dissolved in a solution of LiCl in DMAc. This solvent is thought to work through the disruption of the normally recalcitrant intramolecular H-bonding in cellulose by introducing strong H-bonds to Cl^–^ ions, which are balanced by Li^+^-coordinated DMAc molecules.^36^ MCC was selected for this study over α-cellulose or any variety of pulp as a cellulosic backbone to facilitate rapid, complete dissolution and to maintain a low reaction viscosity. Although this material is structurally analogous to Ac-Dex, we have elected to refer to this polymer by its substituents instead of calling it Ac-Cel both because of the wide range of structural diversity possible within the class of acetal-modified polymers and to avoid confusion with cellulose acetate, modified with acetyl groups. Thus, because it is modified with a mixture of methoxyisopropyl and isopropylidene acetals, the material will be referred to as MiP-Cel. After reacting for 1 h, MiP-Cel was isolated by precipitation into alkaline water. The resulting white powder was found to be soluble in a wide range of organic solvents, including chloroform, DMSO, DCM, EtOAc, and THF. Purification by dissolution in THF and reprecipitation into hexanes yielded pure MiP-Cel as a white powder. GPC analysis reveals a *M*_w_ of 135 kDa and that the dispersity is 4.4 (Figure S1). Because MCC is produced through partial hydrolysis of cellulose fibers, the high dispersity is to be expected.^18^

**Scheme 1:**
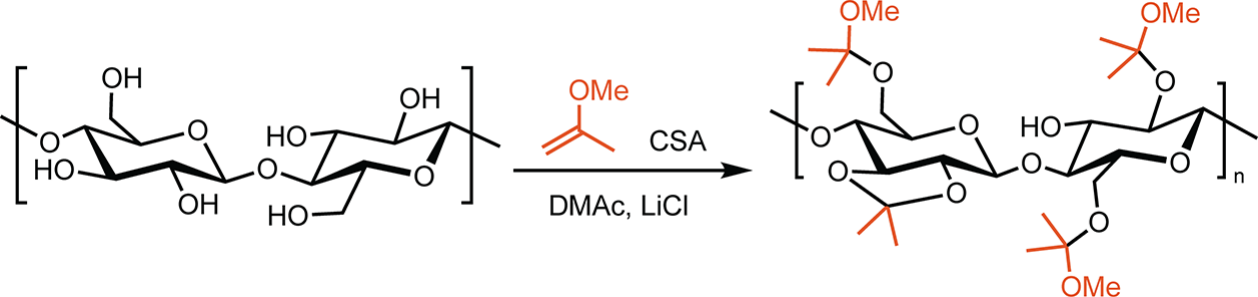
Modification of Cellulose to Form MiP-Cel

Bulk characterization by ATR-FTIR spectroscopy revealed significant changes after modification (Figures 1 and S2). New absorption peaks at 2943 and 2988 cm^–1^ can be attributed to C–H stretching from newly added CH_3_ groups, and absorption at 2832 cm^–1^ supports the installation of new OCH_3_ groups.^37^ The relative decrease in intensity of the OH peak, along with its shift to higher energy from 3324 to 3459 cm^–1^, corresponds to a decrease in both the abundance of hydroxyls and to a reduction of intra- and intermolecularly H-bonding involving the hydroxyls.^38,39^ Acidic hydrolysis of MiP-Cel restores the OH peak to 3323 cm^–1^ and eliminates the new alkyl absorptions, supporting the regeneration of cellulose upon degradation. We attribute the increased broadness of the OH peak to a greater distribution of hydrogen bonding strengths, potentially indicating the altered crystallinity of the regenerated cellulose.

**Figure 1:**
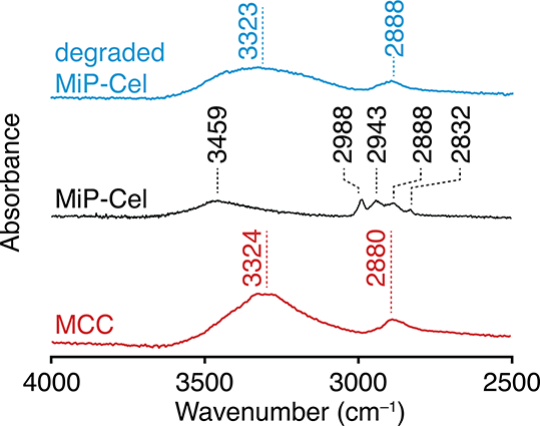
X–H region of ATR-FTIR spectra comparing MCC, MiP-Cel, and the product of MiP-Cel degradation.

The disruption of crystal packing upon modification can also be observed using powder X-ray diffraction (XRD, Figure 2). MCC is known to exist primarily as cellulose I and has characteristic 2θ peaks at 15.1°, 16.8°, 21.1°, 23.0°, and 34.9° corresponding to Miller indices of 110, 11̅0, 200, and 004.^40,41^ After modification, these reflections are not observed, and MiP-Cel appears to be mostly amorphous, with broad peaks centered at 8.7° and 17.7°. These correspond to the first and second reflections of an average interatomic distance of 10.1 Å, which is the length of a single cellobiose along the polymer axis.^42^ Together, these data support the disruption of intermolecular hydrogen bonding and the conversion of crystalline MCC into an amorphous material. Consistent with the formation of regenerated cellulose, degradation of MiP-Cel produces an XRD diffractogram consistent with cellulose II, featuring peaks at 12.4°, 19.7°, and 21.9° corresponding to 11̅0, 110, and 020 planes.^41^ This outcome supports the hypothesized mechanism of material degradation, where pure cellulose is generated after acetal hydrolysis. The production of cellulose II through regeneration from solution has previously found widespread application in the form of cellophane and textiles like rayon, lyocell, and modal.^43^ That MiP-Cel degradation also produces cellulose II suggests possible promise for a new method of regenerated cellulose fiber production with potentially new material properties.

**Figure 2:**
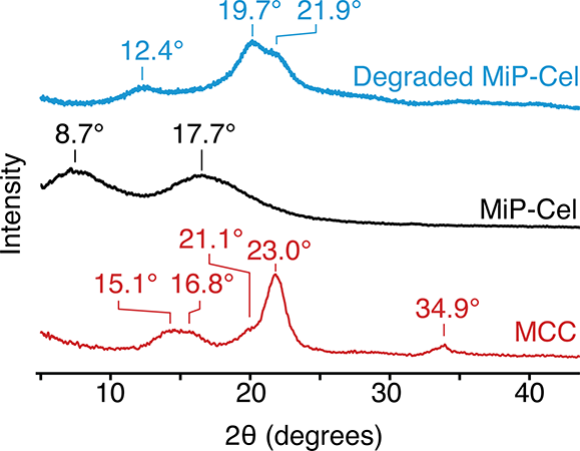
Powder XRD diffractogram comparing MCC, MiP-Cel, and degraded MiP-Cel.

Because of its increased solubility in organic solvents, MiP-Cel could be subsequently characterized by NMR spectroscopy (Figures 3 and S3). ^1^H NMR revealed that all resonances are significantly broadened, as expected for a polymer with randomly grafted substituents. In addition to the anomeric and ring protons, new resonances are observed at δ_H_ 3.1 and 1.3 ppm corresponding to methoxy groups and isopropylidene acetals, respectively. Integration of these regions allowed the determination of acetal content in MiP-Cel to be DS_acyclic_ = 0.88 and DS_cyclic_ = 0.57, indicating that 67% of hydroxyls are modified (Table 1).

**Figure 3:**
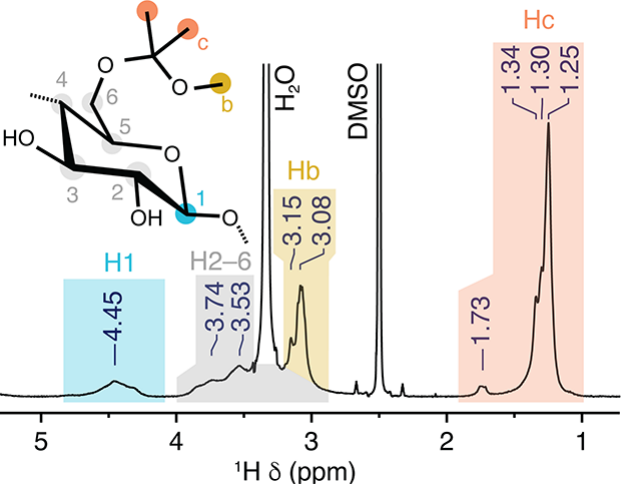
^1^H NMR analysis of MiP-Cel shows the expected anomeric protons, ring protons, methoxy groups, and methyl groups.

**Table 1:** Summary of MiP-Cel Substitution Analyses

	DS acyclic	DS cyclic	% modification
^1^H NMR	0.88	0.57	67
^13^C NMR	0.91	0.53	66
degradation	0.83	0.45	58
average	0.9 ± 0.1	0.5 ± 0.1	64 ± 5

Further corroborating evidence for the degree of modification was collected through the analysis of MiP-Cel hydrolysis byproducts. MiP-Cel was suspended in D_2_O containing DCl and an internal standard. As expected, the acidic hydrolysis reaction generated methanol and acetone; although these could not be directly compared to the concentration of cellulose, a comparison with the internal standard allowed calculation of the acetal content that would have yielded the observed amounts of methanol and acetone (Figure S4). These calculations indicate DS_acyclic_ = 0.83 and DS_cyclic_ = 0.45, indicating that 58% of the hydroxyls are modified (Table 1). This is in general agreement with the calculation based on ^1^H NMR; the slight reduction in observed modification might in part be due to the evaporative loss of methanol and acetone from solution as vapor pressure is established within the sealed NMR tube.

Final corroboration of these measurements could be accomplished by using quantitative ^13^C NMR (Figures 4 and S5). This was accomplished through the use of Cr­(acac)_3_ as a T1 relaxing agent.^44^ The increased dispersion in chemical shifts allows for the identification and quantification of multiple varieties of linear and cyclic acetals. Two primary resonances at δ_C_ 47.9 and 49.1 ppm correspond to linear acetals. Based on the relative reactivity of the hydroxyls on cellulose,^45,46^ these are likely to correspond to modification of the hydroxyls on C6 and C2. Peaks between 24 and 28 ppm δ_C_ correspond to methyl groups on isopropylidine acetals. Because these groups can correspond to linear or cyclic acetals in multiple positions, and because the methyl groups are diastereotopic, it was not possible to assign peaks within this region with confidence. Similarly, the overlap of anomeric peaks and ketal carbon peaks in the δ_C_ 97–103 ppm range prevents assignment. Nonetheless, the areas under these curves support a picture of MiP-Cel where cellulose is modified with a roughly 3:2 ratio of acyclic methoxyisopropylidene acetals and cyclic isopropylidiene acetals. A summary of the three methods of analyzing composition is shown in in Table 1. Studies on acetal-modified dextran have shown that variable degrees of modification are possible by quenching the reaction at early time points.^27^ This variability is greatly reduced at extended reaction times, and reproducible composition was achieved by quenching after 1 h. Comparison of 8 separate batches revealed total acetal content to be consistent, with only 4% variability between samples as measured by ^1^H NMR.

**Figure 4:**
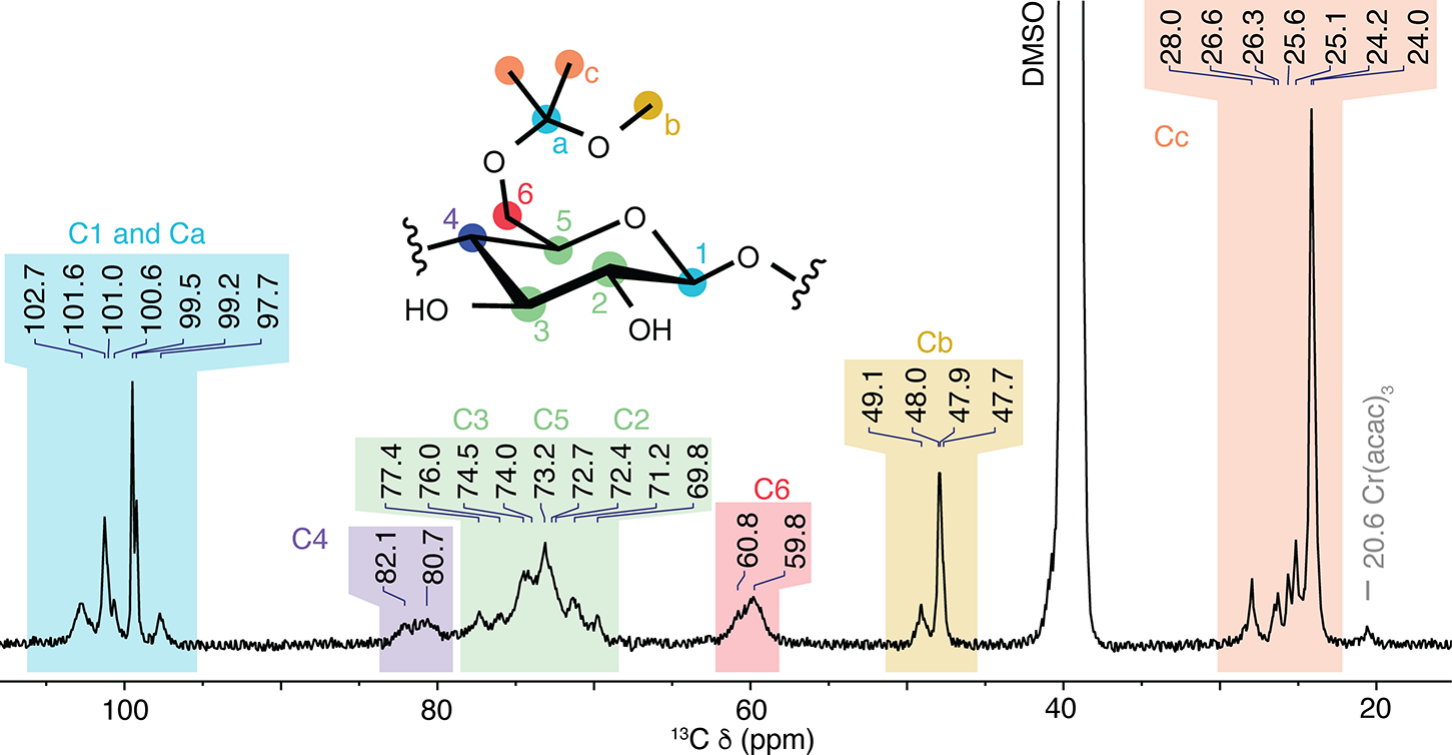
Quantitative 13C NMR of MiP-Cel.

Freestanding transparent films of MiP-Cel could be easily formed through drop-casting techniques (Figure 5a). The transparency of a 12 μm film was found to be 96.6 ± 0.7% averaged across three samples and over the visible spectral range of 380–740 nm (Figure 5b).^47^ Contact angle analysis confirmed that the material is relatively hydrophobic with an advancing contact angle of 98° (Figure 5c). A receding contact angle of 60° leads to a moderately large hysteresis angle of 38°, indicating that water significantly wets the surface of drop-cast MiP-Cel films.^48^ Films appeared level and continuous when inspected by SEM (Figure 5d). While mostly smooth, divots could be observed with a diameter of 1.2 ± 0.4 μm and a density of 7 × 10^3^ instances/mm^2^. Optimization of the drop-casting temperature and starting concentration may potentially further improve microscopic smoothness. Similar to other nonplasticized cellulose-based materials, MiP-Cel films were found to be relatively brittle, with a tensile strength of 40 MPa at 3.6% elongation, with a Young’s modulus of 2 GPa (Figure S5).^49^

**Figure 5:**
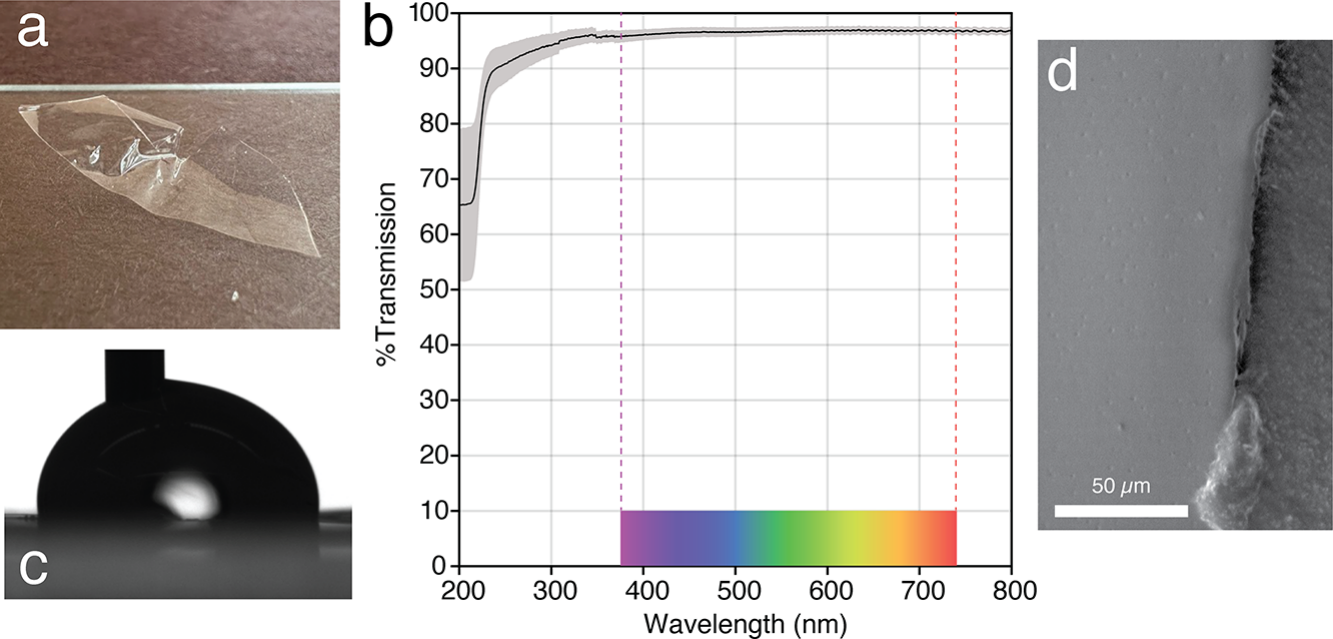
Analysis of MiP-Cel films. (a) Freestanding MiP-Cel film. (b) Optical transparency of a 12 μm thick film with visual spectral range annotated. (c) An advancing contact angle measured as 98°. (d) SEM micrograph of film (left) on carbon tape (right).

The bulk degradability of MiP-Cel films was next measured to assess the potential of these materials as biodegradable plastics. Films were prepared and supported on glass coverslips, and mass loss was observed during continuous submersion in neutral or acidic aqueous conditions (Figure 6a). When submerged in a pH 5 buffer, mass decreased to 36% of the initial mass over the course of 4 days, attributable to the hydrolysis of ketal functionalities and significant loss of film integrity. XRD and IR analyses of the degraded materials indicate that the remaining material is regenerated cellulose (Figures 1 and 2). In contrast, films submerged in pH 7.4 maintained this mass over the same time period. There is an increase in mass over the first day, which, in line with the high degree of wetting seen in contact angle experiments, can be attributed to absorption of water into the films. After 12 days at neutral pH, 74 ± 7% of the initial mass still remained. The remaining films were observed to still be significantly hydrophobic, and the lost mass can be attributed to the combination of mechanical losses in the film isolation process and slow hydrolysis of ketal functionalities. This pH-dependent change in film stability highlights one avenue for intentional degradation.

**Figure 6:**
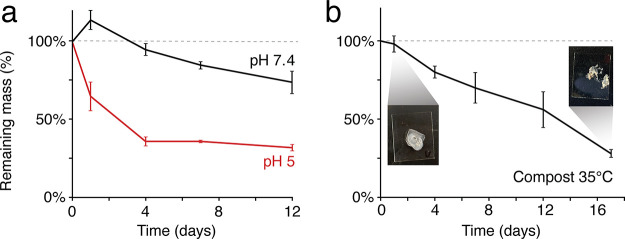
Degradation of MiP-Cel films in buffer (a) or compost (b). Insets show a loss of mechanical integrity over the course of compost incubation.

Composting may be a more desirable mode of waste remediation than targeted acidic degradation. Many bioplastics require industrial composting to be completely biodegradable, and incomplete degradation can lead to the release of undesirable microplastics.^50,51^ Thus, we evaluated the viability of home composting as a method for MiP-Cel degradation (Figure 6b). Films were submerged in home compost and incubated at 35 °C, and their mass was monitored over time. Mass decreases in a roughly linear fashion over 17 days to 23 ± 3% of initial mass. Because of the loss of mechanical integrity, the experiment was discontinued at this point. The remaining material is expected to be cellulose. The high extent of material degradation observed indicates that MiP-Cel-based materials are viable candidates to be fully compostable.

## 4 Conclusions

We have identified that cellulose can be modified with methoxy isopropylidene ketals to form MiP-Cel, a hydrophobic material with good solution processability that can be degraded back into cellulose under environmentally accessible conditions. The material is prepared quickly and easily in a single chemical transformation using affordable and widely available materials. MiP-Cel is an amorphous polymer with a high overall degree of modification with a 3:2 mixture of acyclic methoxy isopropylidine and cyclic isopropylidine ketals. While cyclic ketals fit well with data from NMR and degradation analysis, the specific modification pattern could not be assessed. It is possible that cyclic ketals comprise a mixture of regiochemistries spanning both within a single AGU and between adjacent AGUs. GPC analysis demonstrates that int*er*strand acetals are unlikely to exist in large numbers.

Although MiP-Cel is chemically very similar to the acetal-modified dextran Ac-Dex, only MiP-Cel can be used to easily prepare free-standing solution-cast films. This may be, in part, because conversion from a α-(1→6) linkage to a β-(1→4) linkage imparts a significant change to the geometry of the backbone. Additionally, dextran possesses approximately 5% α -(1→3) branching, which may disrupt packing in the solid state relative to unbranched cellulose derivatives.^52^ The ability of MiP-Cel to produce films suggests that it might be suitable as the basis for other macroscopic materials, such as fibers or membranes.

We find that MiP-Cel can be degraded into regenerated cellulose under mild conditions and that it can be composted under nonindustrial conditions. Further characterization and optimization of thermal, mechanical, and barrier properties will be necessary to identify the viability of MiP-Cel in particular, in packaging or agricultural contexts. In addition to the potential applications of this specific material, these findings demonstrate the potential viability of ketal-type acetals as the basis for new highly biodegradable bioplastics.

## Supplementary Material



## Notes

GPC analysis of MiP-Cel, uncropped FTIR spectra, ^1^H and ^13^C NMR spectra of MiP-Cel, ^1^H NMR of degraded MiP-Cel for substitution analysis, and substitution calculation details (PDF)

## †

University of Auckland, Auckland 1142, New Zealand

## Abbreviations

PLA: poly­(lactic acid)
MCC: microcrystalline cellulose
acac: acetylacetonate
DS: degree of substitution
AGU: anhydroglucose unit
DMAc: dimethylacetamide
NMR: nuclear magnetic resonance
CSA: camphorsulfonic acid
THF: tetrahydrofuran
DMSO: dimethylsulfoxide
GPC: gel permeation chromatography
ATR-FTIR: attenuated total reflectance Fourier transform infrared
XRD: X-ray diffraction
DSS: 4,4-dimethyl-4-silapentane-1-sulfonic acid
