# Supplementary material for: Ketal-Modified Cellulose as a Biodegradable Bioplastic
Source: ACS Omega. 2025 Nov 14;10(46):56025–31. doi: 10.1021/acsomega.5c07631 (PMC12658691; doi:10.1021/acsomega.5c07631)
Supplement: Supplementary file 1 [file ao5c07631_si_001.pdf]

Supporting Information

Ketal-Modified Cellulose as a Biodegradable Bioplastic

Kyle E. Broaders[*], Elizabeth Kuehne, Abigail C. Bowden, Maxine R. Fraser

[1]Department of Chemistry, Mount Holyoke College, South Hadley, Massachusetts 01075, USA

Corresponding Author

*E-mail: broaders@mtholyoke.edu

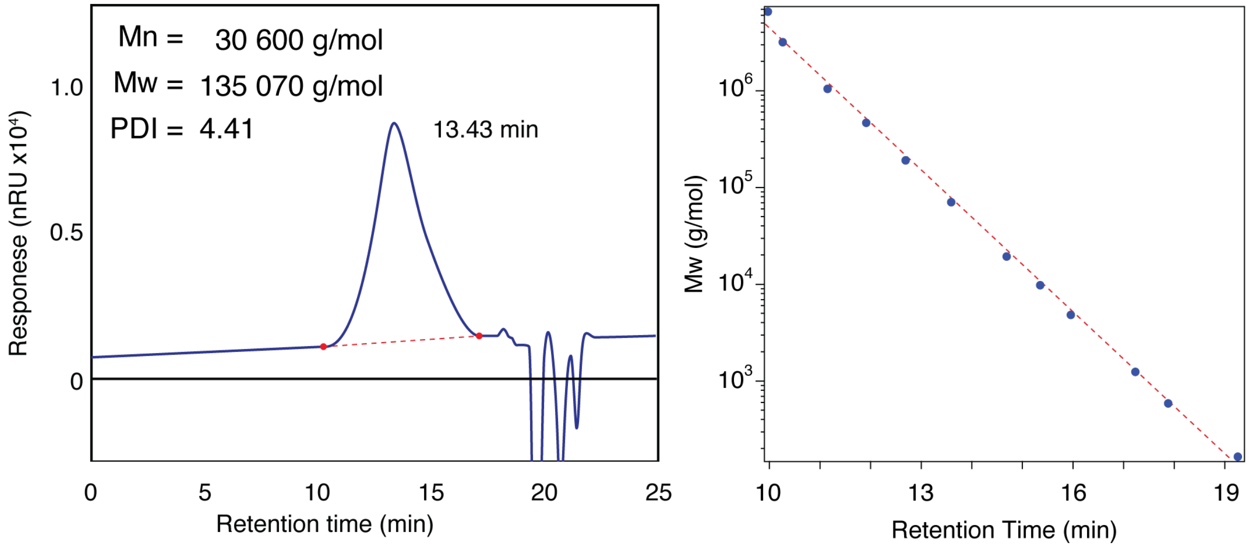

Figure S1: GPC analysis was conducted with an Agilent 1260 Infinity II system with two PLgel 5 µm Mixed-C columns connected in parallel. Separation and analysis were conducted at a flow rate of 1 mL/min in HPLC grade THF at 35 °C with a refractive index detector. Chromatographs were calibrated with a poly(styrene) standard (right) and are uncorrected.

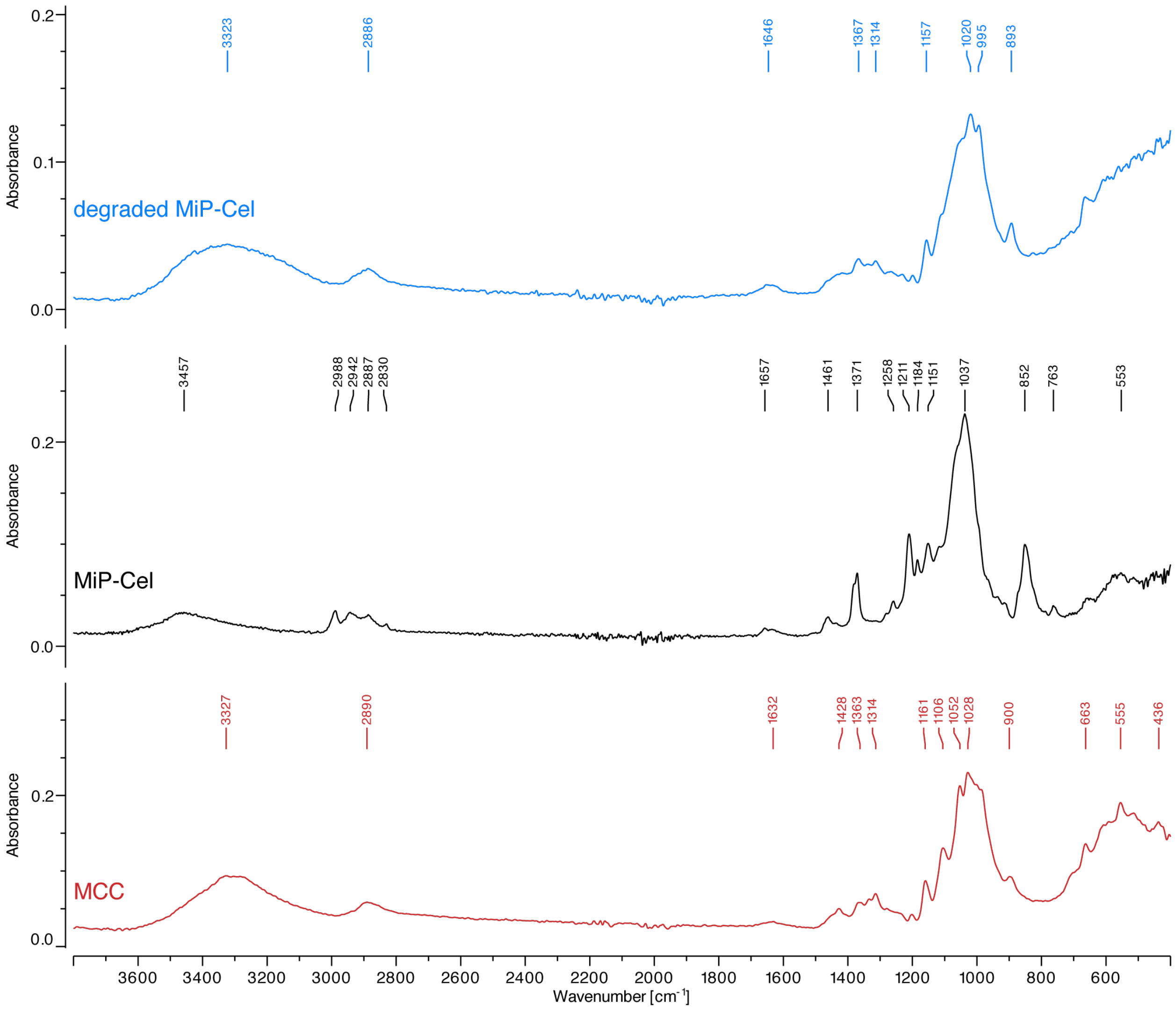

Figure S2: Full ATR-FTIR spectra of MCC (bottom, red), MiP-Cel (middle, black), and MiP-Cel degraded under acidic aqueous conditions (top, blue).

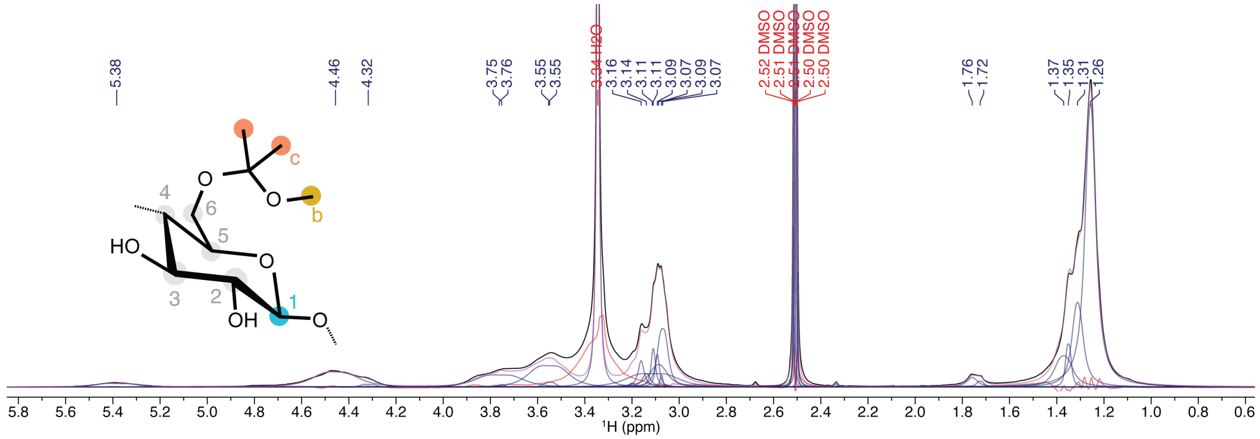

Figure S3: Line fitting analysis of ^{1}H NMR of MiP-Cel. Original data (black) was decomposed into underlying peaks (navy) using MestReNova. Sum of fit peaks is represented in magenta and residual error is represented in red.

Table S1: Assignment of fit peaks ^{1}H NMR of MiP-Cel. Ring protons were left unassigned and solvent was omitted for clarity. Relative integrations summarized on the right table and converted to molar ratios below.

#	ppm	Height	Width	L/G	Area	Assign
1	1.257	1998.1	21.4	0.43	509142	Hc
2	1.312	598.6	22.2	0.61	157354	Hc
3	1.351	304.8	11.3	1.41	36068	Hc
4	1.371	220.6	34.9	1	74726	Hc
5	1.724	43.6	14.5	0.82	7112	Hc
6	1.758	68.4	21.2	0.08	17898	Hc
7	3.070	413.7	20.7	1.31	92042	Hb
8	3.075	84.2	5.7	0.57	6247	Hb
9	3.088	159.0	35.4	0.53	67206	Hb
10	3.093	230.9	8.4	0.33	24055	Hb
11	3.110	271.9	8.8	0.3	29754	Hb
12	3.114	97.2	100.0	2	126411	
13	3.139	93.1	16.7	0.46	20556	Hb
14	3.161	186.5	11.9	0.07	28544	Hb
15	3.200	28.6	10.2	1.47	2735	
16	3.345	5069.1	4.5	0.01	297616	
17	3.549	35.8	22.4	1.92	7661	
18	3.553	152.8	77.5	2	111331	
19	3.752	18.9	12.0	1	2515	
20	3.763	86.9	92.6	2	75660	
21	4.318	20.9	28.2	2	5555	H1
22	4.457	109.7	87.2	1.43	85664	H1
23	5.384	26.9	79.9	2	20239	OH

Assign	Total Area	Rel. Int.
H1	91219	1.00
Hb	268404	2.94
Hc	802301	8.80

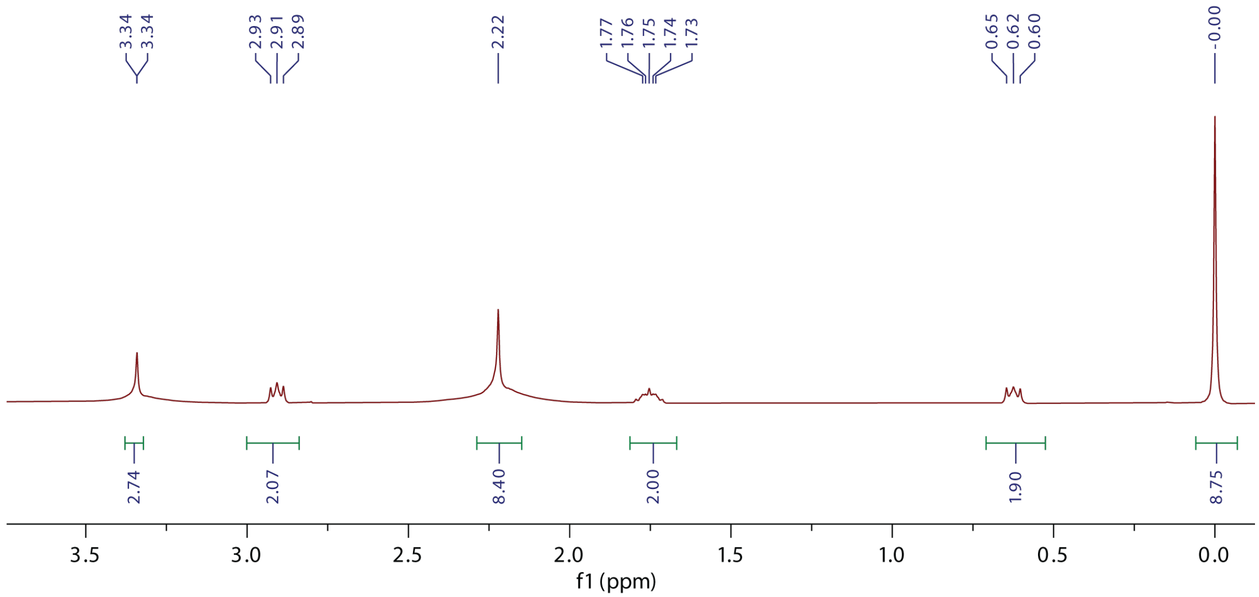

Figure S4: NMR of methanol and acetone generated by degradation of a known amount of MiP-Cel in D_2O containing DCl along with 100 mM 4,4-dimethyl-4-silapentane-1-sulfonic acid (DSS) as a standard.

Acetal composition as measured by degradation was calculated using the following equations:

$$DS_{total} = \frac{mol\ acetone}{mol\ cellulose\ AGU}$$

$$DS_{acylic} = \frac{mol\ MeOH}{mol\ cellulose\ AGU}$$

$$DS_{total} = DS_{acyclic} + DS_{cyclic}$$

Because cellulose is not soluble in D_2O, the amount of cellulose was calculated after accounting for the mass contributions of acetals from the starting the polymer mass:

$$mass\ MiP\text{-}cel = mass\ cellulose + \frac{42.09\ g}{mol} * mol\ acetone + \frac{31.03\ g}{mol} * mol\ MeOH$$

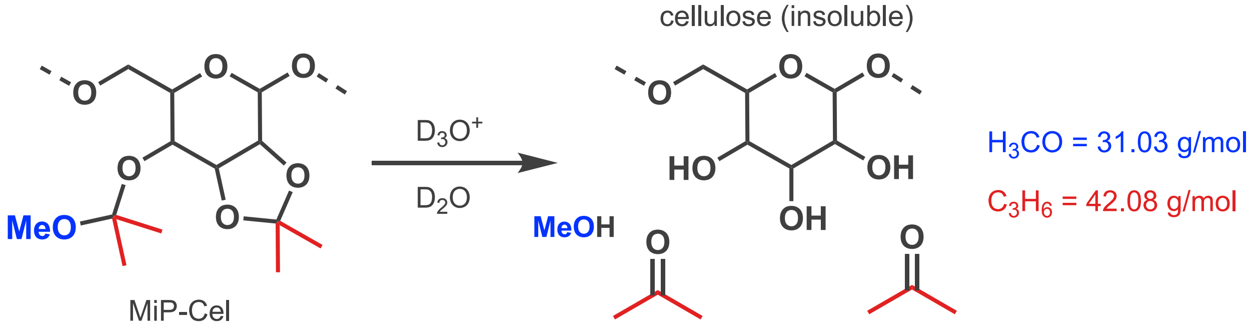

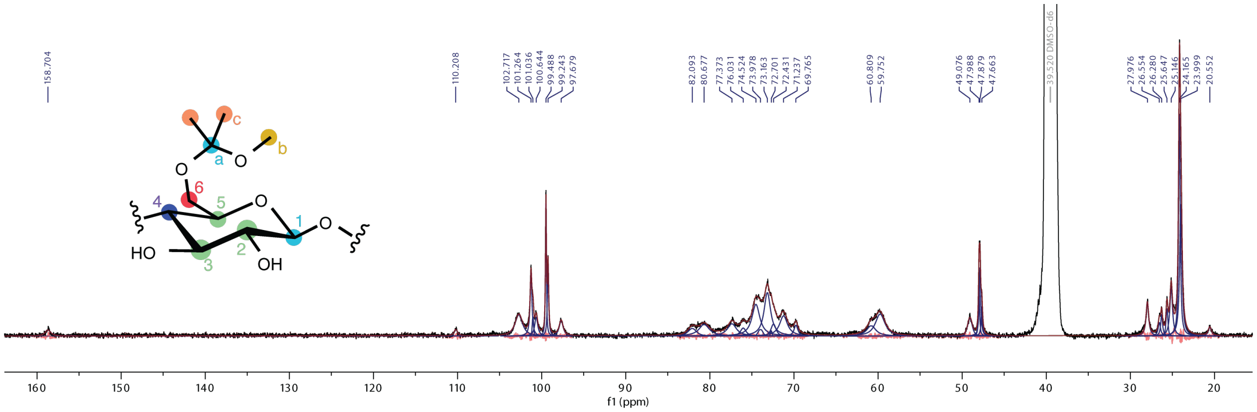

Figure S5: Line fitting analysis of ^{13}C NMR of MiP-Cel. Original data (black) was decomposed into simulated peaks (navy) using MestReNova. Sum of fit = magenta, residual error = red.

Table S2: Assignment of fit peaks ^{13}C NMR of MiP-Cel. Solvent omitted for clarity. Relative integrations summarized on the right table.

peak	ppm	Height	Width	L/G	Area	Assign
1	21	2018	45.7	1.0	197515	Cr(acac)3
2	24	29961	33.4	0.9	2078804	Cc
3	24	50526	23.8	0.8	2421915	Cc
4	25	11171	37.0	1.0	885189	Cc
5	26	6274	25.9	0.5	291159	Cc
6	26	4953	26.3	1.0	278526	Cc
7	27	3109	33.6	1.0	223750	Cc
8	28	7657	36.9	1.0	604971	Cc
9	48	6726	19.7	1.0	283827	Cb
10	48	15532	21.1	0.9	688177	Cb
11	48	11827	15.5	0.8	366590	Cb
12	49	4039	66.0	0.5	481613	Cb
13	60	5005	137.0	0.9	1408747	C6
14	61	2417	129.7	1.0	671258	C6
15	70	2484	55.0	0.9	284568	C2
16	71	4614	114.5	1.0	1131094	C2
17	72	2747	71.0	0.7	382696	C2
18	73	1243	20.1	0.3	40646	C2
19	73	9886	94.9	1.0	1998683	C5
20	74	1562	64.9	0.0	147118	C3
21	75	7228	113.5	1.0	1743008	C3
22	76	1837	80.4	0.9	308428	C3
23	77	2825	128.9	1.0	779718	C4
24	81	2675	150.0	1.0	859105	C4
25	82	1741	119.7	0.7	395444	C4
26	98	3517	56.6	0.9	412768	Ca
27	99	16142	18.1	0.9	604892	Ca
28	99	30084	13.4	0.8	803586	Ca
29	101	4354	42.8	1.0	398845	Ca
30	101	4370	19.8	0.0	125690	Ca
31	101	14488	25.3	1.0	783500	C1
32	103	4867	106.7	0.9	1059231	C1
33	110	1516	51.0	1.0	165414	Cr(acac)3
34	159	1390	81.2	1.0	241517	Cr(acac)3

Assign	Total Area	Rel. Int.
C1	1842731	0.92
C2	1839003	0.92
C3	2198554	1.10
C4	2034266	1.02
C5	1998683	1.00
C6	2080005	1.04
Ca	2345781	1.17
Cb	1820206	0.91
Cc	6784315	3.39

Calculation of degree of substitution (DS) by ^{1}H NMR and ^{13}C NMRwas carried out by finding the molar ratio of components of MiP-Cel followed by use of the following equations.

$$DS_{total} = \frac{mol\ isopropylidene}{mol\ cellulose\ AGU}$$

$$DS_{acylic} = \frac{mol\ methoxy}{mol\ cellulose\ AGU}$$

$$DS_{total} = DS_{acyclic} + DS_{cyclic}$$

Example calculations:

For ^{1}H NMR:

Assign	Component name	Rel. Int.	Protons represented	Molar Ratio
H1	cellulose AGU	1.00	1	1.00
Hb	methoxy	2.94	3	0.98
Hc	isopropylidene	8.80	6	1.47

Thus: $DS_{total} = 1.47$ $DS_{acyclic} = 0.98$ $DS_{cyclic} = 0.49$

For ^{13}C NMR:

Assign	Component name	Rel. Int.	Carbons represented	Molar ratio
C1	cellulose AGU	0.92	1	0.92
C2	cellulose AGU	0.92	1	0.92
C3	cellulose AGU	1.10	1	1.10
C4	cellulose AGU	1.02	1	1.02
C5	cellulose AGU	1.00	1	1.00
C6	cellulose AGU	1.04	1	1.04
Ca	isopropylidene	1.17	1	1.17
Cb	methoxy	0.91	1	0.91
Cc	isopropylidene	3.39	2	1.70

Averaging molar ratios, $DS_{total} = 1.44$ $DS_{acyclic} = 0.91$ $DS_{cyclic} = 0.53$

Percent hydroxyl coverage is calculated using:

$$\%OH\ covered = \frac{DS_{acyclic} + 2\ DS_{cyclic}}{3} \times 100$$

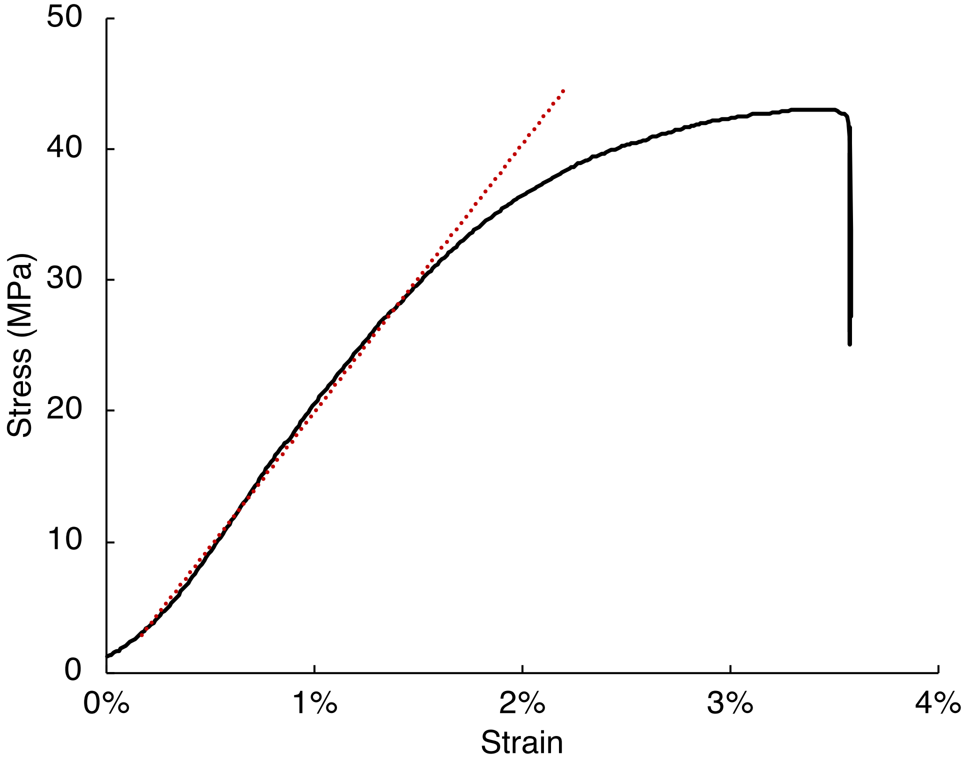

Figure S5: Tensile testing of MiP-Cel film showing a tensile strength of 43 MPa at 3.6% elongation. Estimated linear region fitting highlighted as dotted red line indicating Young's modulus of 2.0 GPa.
